# Supplementary material for: Mutant FUS causes DNA ligation defects to inhibit oxidative damage repair in Amyotrophic Lateral Sclerosis
Source: Nat Commun. 2018 Sep 11;9:3683. doi: 10.1038/s41467-018-06111-6 (PMC6134028; doi:10.1038/s41467-018-06111-6)
Supplement: Supplementary file 1 — Supplementary Information [file 41467_2018_6111_MOESM1_ESM.pdf]

Supplementary Information

Mutant FUS causes DNA ligation defects to inhibit oxidative damage repair in Amyotrophic Lateral Sclerosis

Haibo Wang[1], Wenting Guo[2,3], Joy Mitra[1], Pavana M. Hegde[1], Tijs Vandoorne[2,3], Bradley J. Eckelmann[1,4], Sankar Mitra[1,5], Alan E Tomkinson[6], Ludo Van Den Bosch[2,3] and Muralidhar L. Hegde[1,5,7,*]

[1]Department of Radiation Oncology, Houston Methodist Research Institute, Houston, Texas 77030, USA

[2]KU Leuven-Department of Neurosciences, Experimental Neurology and Leuven Brain Institute (LBI), Leuven, Belgium

[3]VIB, Center for Brain & Disease Research, Laboratory of Neurobiology, Leuven, Belgium

[4]Texas A&M Health Science Center, College of Medicine, Bryan, TX 77807, USA

[6]Departments of Internal Medicine, and Molecular Genetics and Microbiology and University of New Mexico Comprehensive Cancer Center, University of New Mexico, Albuquerque, New Mexico, USA

[5]Weill Medical College, New York, USA

[7]Houston Methodist Neurological Institute, Institute of Academic Medicine, Houston Methodist, Houston, Texas, USA

*Correspondence and requests for materials should be addressed to M.L.H. (email: mlhegde@houstonmethodist.org)

Supplementary Figures

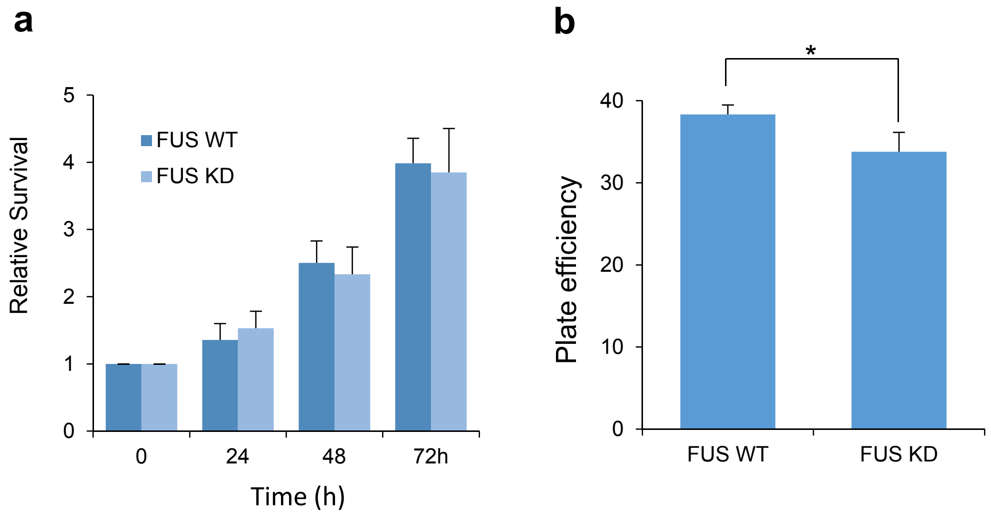

Supplementary Figure 1. Effect of FUS KD on the steady state cell viability and proliferation. (a) MTT based viability analysis of FUS WT and FUS KD SH-SY5Y cells performed at indicated time points.

(b) Clonogenic survival analysis of FUS WT and FUS KD HEK293 cells. All error bars are standard deviation of experiments performed in triplicate (*, $p<0.05$, two-tailed unpaired Student's t-test).

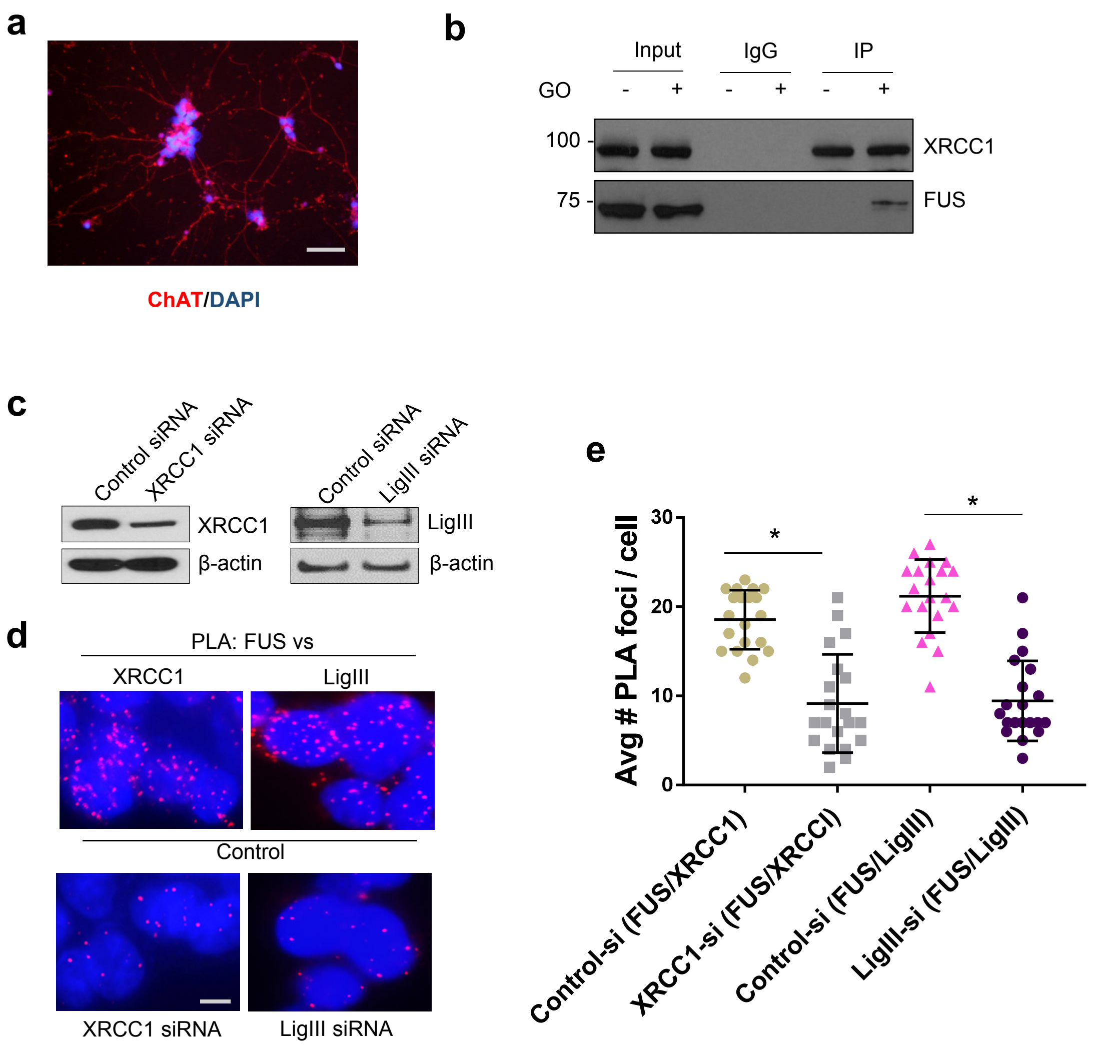

Supplementary Figure 2. Association of FUS and XRCC1 in human iPSC-derived motor neurons.
(a) IF staining of motor neurons differentiated from healthy individual derived iPSC lines for differentiation marker ChAT. Nuclei are stained with DAPI. Scale bar = 50 µm.
(b) IB of endogenous XRCC1 co-IP from SH-SY5Y cells for FUS.
(c) IB showing XRCC1 and LigIII KD by siRNAs. Total lysate were extracted from SH-SY5Y cells 48h after the transfection. β-actin was probed as loading control.
(d) PLA of FUS vs XRCC1 or LigIII in XRCC1 or LigIII KD in GO-treated SH-SY5Y cells. Nuclei stained with DAPI. Scale bar = 5µm.
(e) Quantitation of PLA foci from 25 cells. The error bars are standard deviation of experiments performed in triplicate (*, $p<0.05$, two-tailed unpaired Student's t-test).

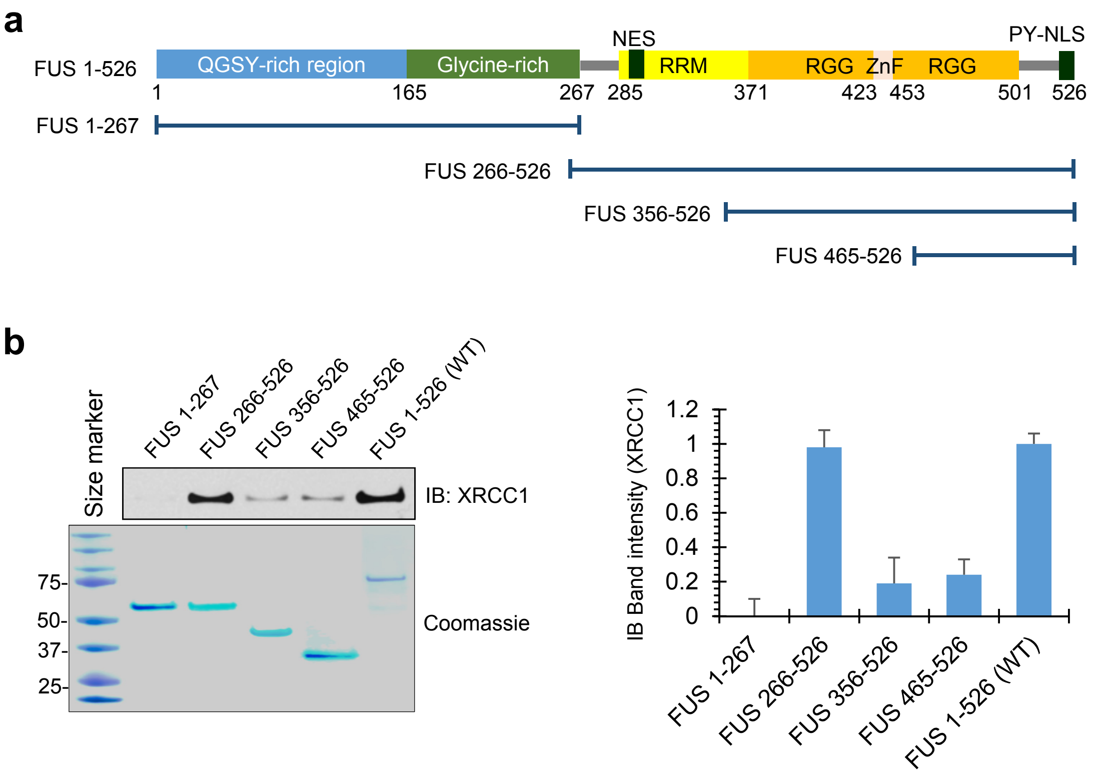

Supplementary Figure 3. Domain mapping analysis of FUS-XRCC1 interaction.
(a) Schematic of FUS domain polypeptides purified for interaction mapping analysis.
(b) *In vitro* affinity co-elution of various purified GST-FUS fragments with XRCC1. XRCC1 was detected by IB (top panel). Comparable level of FUS polypeptides in the reaction was confirmed by Coomassie staining (bottom panel). The Hisogram on the right shows quantitation of XRCC1 band intensity. The error bars are standard deviation of experiments performed in triplicate.

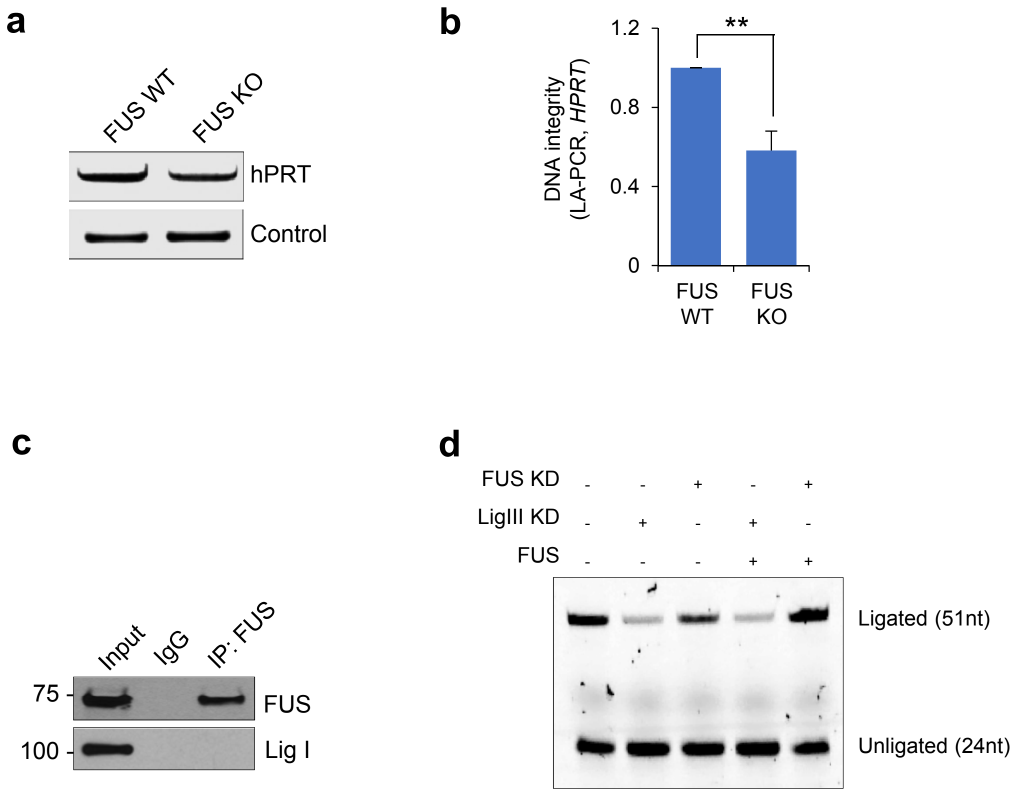

Supplementary Figure 4. Genomic DNA integrity in CRSPR/Cas9 mediated FUS KO cells.
(a) and (b) Integrity of genomic DNA isolated from HEK293 FUS WT or FUS knockout (KO) cells measured by long amplicon quantitative PCR (LA-PCR) analysis. 10.4 kb fragment including exons 2-5 of the hprt gene was amplified and separated in 1% agarose gel. The amplified DNA product was quantified using pico green fluorescence (b). The error bars are standard deviation of experiments performed in triplicate (**, $p<0.01$, two-tailed unpaired Student's t-test).
(c) IB of endogenous FUS co-IP from SH-SY5Y cells for LigI. The IP was performed with anti-FUS antibody. (d) *In vitro* DNA nick ligation activity assay. XRCC1 IP complex from nuclear extract of GO treated FUS WT, FUS KD and LigIII KD SH-SY5Y cells was incubated with or without purified FUS.

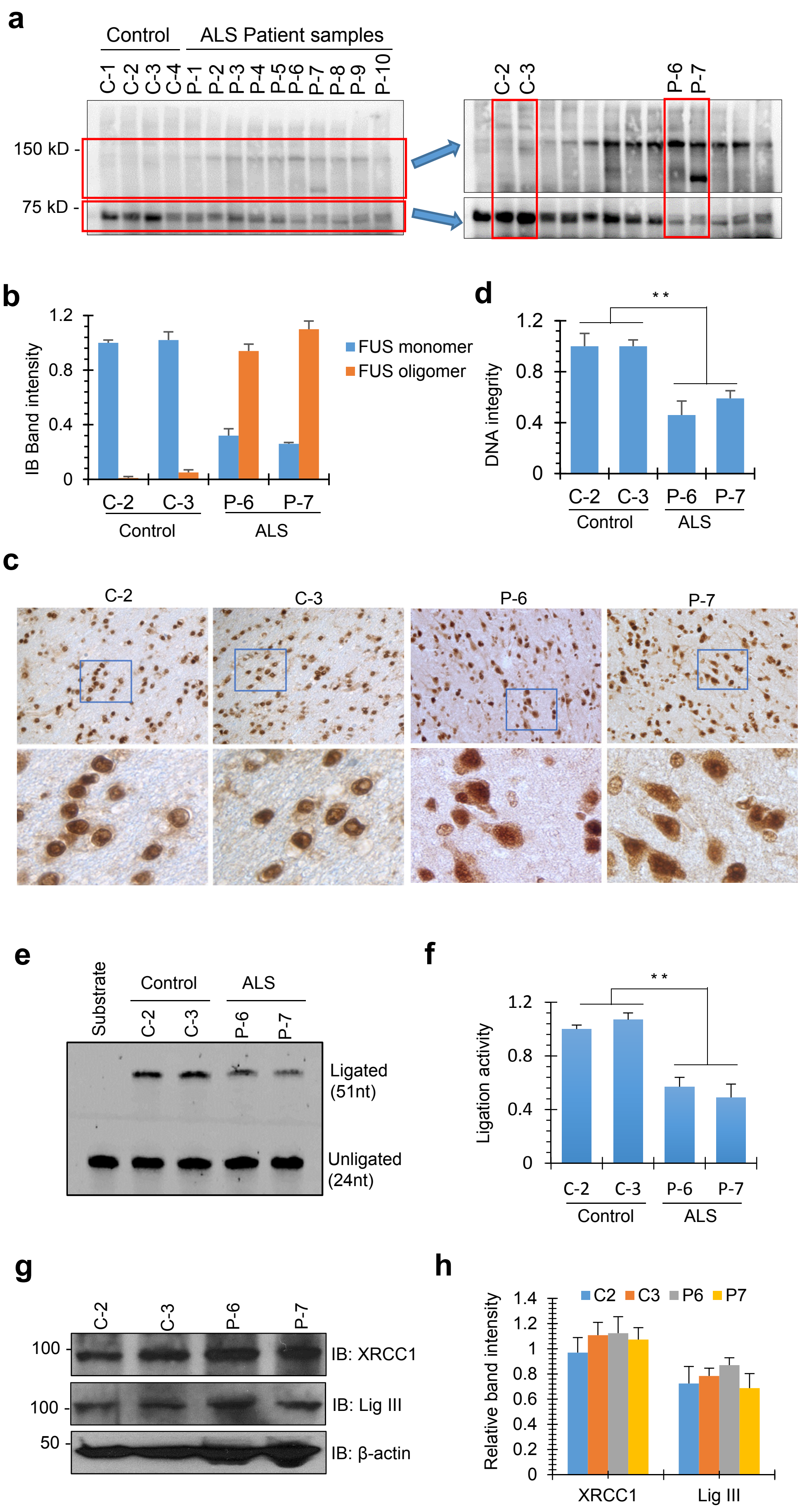

a
Control
ALS Patient samples
C-1 C-2 C-3 C-4 P-1 P-2 P-3 P-4 P-5 P-6 P-7 P-8 P-9 P-10
150 kD -
75 kD -
C-2 C-3 P-6 P-7
b
IB Band intensity
1.2
0.8
0.4
0
FUS monomer
FUS oligomer
C-2 C-3 P-6 P-7
Control
ALS
d
DNA integrity
**
1.2
0.8
0.4
0
C-2 C-3 P-6 P-7
Control
ALS
c
C-2
C-3
P-6
P-7
e
Substrate
Control
ALS
C-2 C-3 P-6 P-7
Ligated (51nt)
Unligated (24nt)
f
Ligation activity
**
1.2
0.8
0.4
0
C-2 C-3 P-6 P-7
Control
ALS
g
C-2 C-3 P-6 P-7
100
IB: XRCC1
100
IB: Lig III
50
IB: β-actin
h
Relative band intensity
C2 C3 P6 P7
1.4
1.2
1
0.8
0.6
0.4
0.2
0
XRCC1
Lig III

Supplementary Figure 5. DNA ligation defect in ALS patients with FUS pathology.

(**a**) and (**b**) IB of spinal cord tissue extracts from control and ALS patients for monomeric and oligomeric FUS. Right image in (a) shown selected area in left image with a longer exposure time. The monomer and aggregated FUS bands were quantitated in (b). Two patients P-6 and P-7, which showed FUS pathology were analyzed for DNA damage and ligation defect analysis.

(**c**) Immunohistochemistry (IHC) showing the localization of FUS in ASL patient derived spinal cord samples.

(**d**) LA-PCR analysis of DNA integrity and quantitation of the products by pico green fluorescence (**, $p<0.01$, two-tailed unpaired Student's t-test).

(**e**) and (**f**) In-vitro DNA nick ligation assay. The control and ALS spinal cord tissue extracts (100ng) were mixed with recombinant XRCC1/LigIII complex (100f.moles) and ligation activity analyzed as before and quantitated (**, $p<0.01$, two-tailed unpaired Student's t-test).

(**g**) and (**h**) IB of spinal cord tissue extract from control and ALS patients for XRCC1 and LigIII. β-actin was probed as a loading control. IB bands intensity is quantified in (g). All error bars are standard deviation of experiments performed in triplicate.

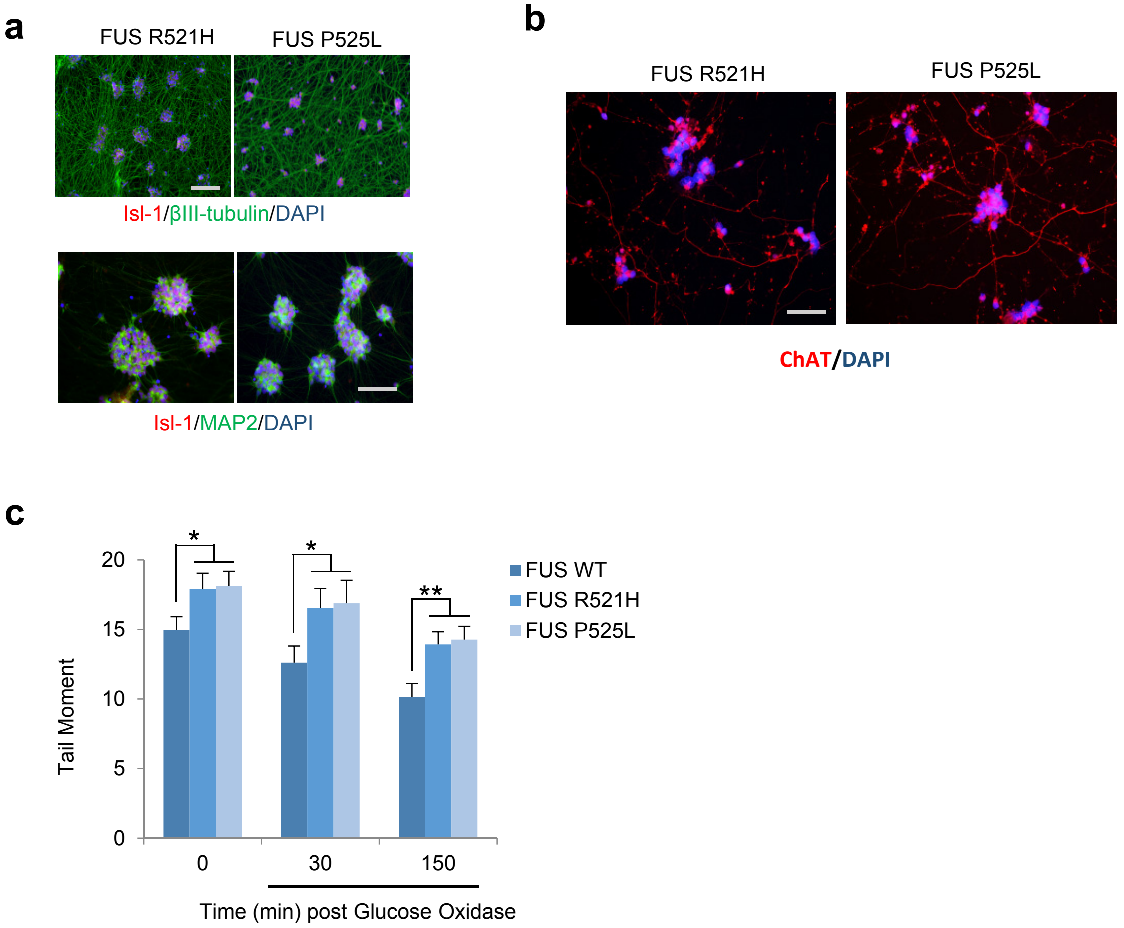

Supplementary Figure 6. FUS mutant iPSC-derived motor neurons from ALS patients fail to repair genome damage.

(a) IF staining of motor neurons differentiated from ALS patient derived iPSC lines for indicated markers proteins. Representative images of motor neurons that stained Isl-1, MAP2 or βIII-tubulin indicated ~80% differentiation efficiency of FUS R521H and FUS P525L mutant iPSCs. Nuclei are stained with DAPI. Scale bar = 50 µm.

(b) IF staining of motor neurons differentiated from ALS patient derived iPSC lines for differentiation marker ChAT. Nuclei are stained with DAPI. Scale bar = 50 µm.

(c) Histogram represent quantitation of mean tail moment from 50 randomly selected nuclei. Alkaline comet assay of motor neurons differentiated from healthy individual and ALS patient derived iPSC lines at 0, 30 and 150 min post GO treatment. The error bars are standard deviation (*, $p<0.05$; **, $p<0.01$, two-tailed unpaired Student's t-test).

a

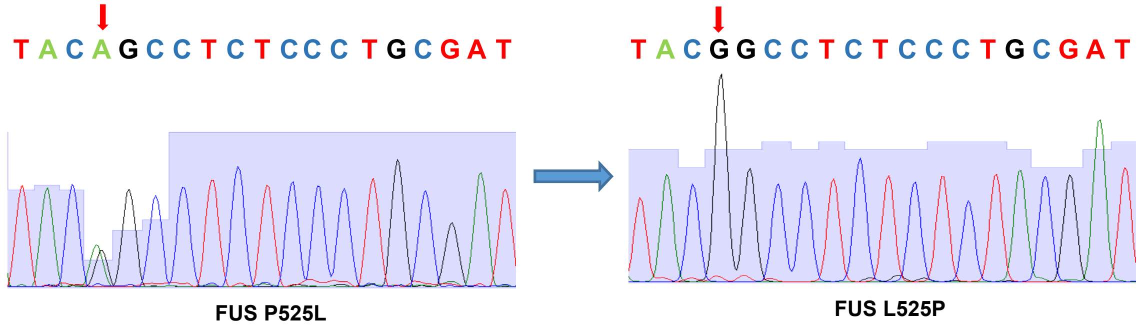

b

Sample Name	Self-renewal	Ectoderm	Mesoderm	Endoderm	ΔSCORES	Self-renewal	Ectoderm	Mesoderm	Endoderm
FUS L525P D0	-2,42	0,88	1,65	-0,25					
FUS L525P D7	-4,51	2,73	3,88	1,39	Co3/1	-2,09	1,85	2,23	1,64

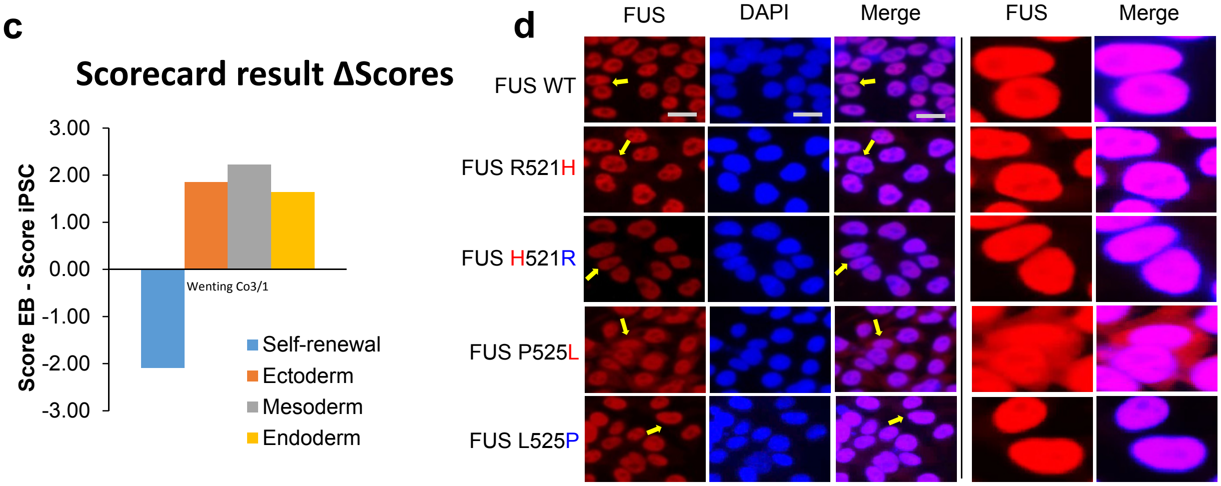

Supplementary Figure 7. Correction of FUS mutations in patient derived iPSC lines rescues DNA ligation defect.

(a) DNA sequencing showing the correction of A to G as indicated by red arrow in P525L iPSC.

(b) and (c) Embryonic body formation analysis showing the presence of the three germ layer markers in L525P iPSCs by using qPCR.

(d) IF analysis showing nuclear clearance of FUS in control and ALS-patient derived iPSC lines with FUS mutations, before and after mutation correction. Scale bar = 50 µm.

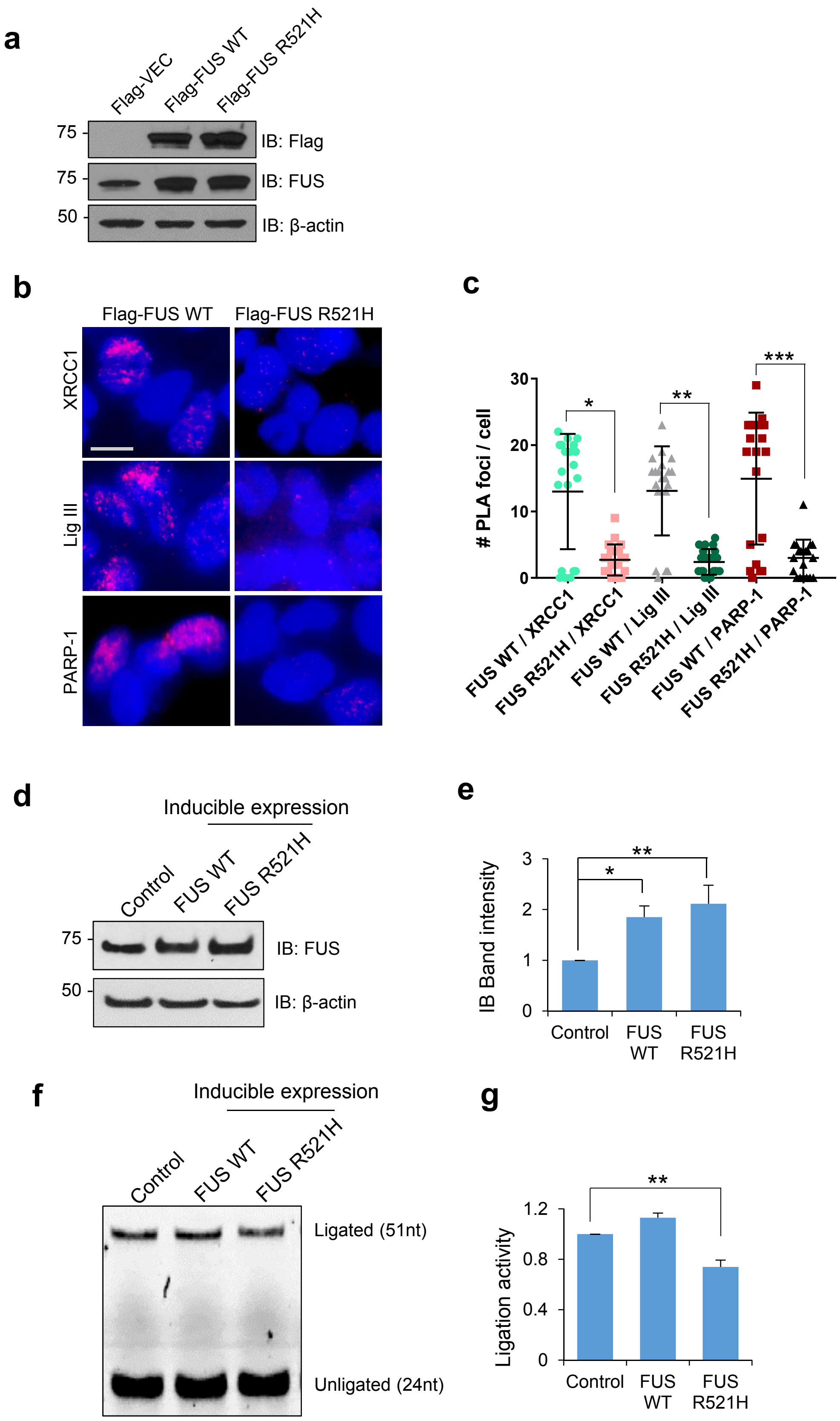

a
Flag-VEC
Flag-FUS WT
Flag-FUS R521H
75
IB: Flag
75
IB: FUS
50
IB: β-actin
b
Flag-FUS WT
Flag-FUS R521H
XRCC1
Lig III
PARP-1
c
PLA foci / cell
30
20
10
0
*
**
***
FUS WT / XRCC1
FUS R521H / XRCC1
FUS WT / Lig III
FUS R521H / Lig III
FUS WT / PARP-1
FUS R521H / PARP-1
d
Inducible expression
Control
FUS WT
FUS R521H
75
IB: FUS
50
IB: β-actin
e
IB Band intensity
3
2
1
0
**
*
Control
FUS WT
FUS R521H
f
Inducible expression
Control
FUS WT
FUS R521H
Ligated (51nt)
Unligated (24nt)
g
Ligation activity
1.2
0.8
0.4
0
**
Control
FUS WT
FUS R521H

Supplementary Figure 8. Defective recruitment and reduced repair complex formation by FUS-R521 mutation causes DNA ligation defect.

(a) IB of ectopically expressed Flag-vector (VEC), Flag-FUS WT and Flag-FUS R521H mutant in
HEK293 cells.

(b) PLA of Flag vs XRCC1, Lig III or PARP1 in Flag-FUS WT or Flag-FUS R521H expressed HEK293
cells following GO treatment. Nuclei are stained with DAPI. Scale bar = 5 μm.

(c) Quantitation of PLA foci from 25 cells of (b). The error bars are standard deviation (**, $p<0.01$. ***, $p<0.001$, two-tailed unpaired Student's t-test).

(d) and (e) IB showing induced FUS WT or FUS R521H overexpression in H9-hESC differentiated motor neurons. The overexpression was induced by adding doxycycline (2μg/ml) in various H9-hESC lines. Histogram shows quantitation of IB band intensity. The error bars are standard deviation of experiments performed in triplicate (*, $p<0.05$. **, $p<0.01$, two-tailed unpaired Student's t-test).

(f) and (g) *In vitro* DNA nick ligation activity assay. XRCC1 IP complex from nuclear extract of motor neurons in (d) and quantified. The error bars are standard deviation of experiments performed in triplicate (**, $p<0.01$, two-tailed unpaired Student's t-test).

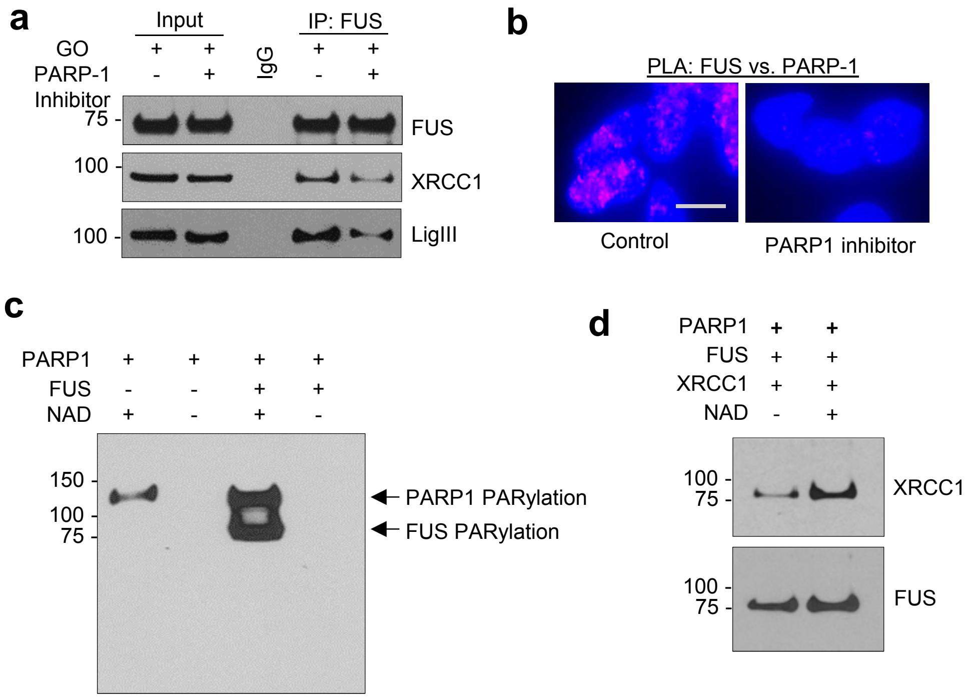

Supplementary Figure 9. PARP-1 activity promotes FUS-XRCC1 interaction.

(a) IB of endogenous FUS co-IP from SH-SY5Y cells for XRCC1 and LigIII, with or without PARP1 inhibitor, AG-14361. The IP was performed with anti-FUS antibody.

(b) PLA of FUS vs PARP1in SH-SY5Ycells following GO treatment, with or without PARP1 inhibitor pre treatment. Nuclei are stained with DAPI. Scale bar = 50 µm.

(c) IB of *in vitro* ADP-ribosylation reaction products under indicated conditions. PARylation was visualized

by probing with anti-Poly (ADP-Ribose) polymer (PAR) antibody.

(d) *In vitro* affinity co-elution of purified GST-FUS with XRCC1, in presence and absence of activated PARP-1. XRCC1 and FUS were detected by IB.

Supplementary Tables

Supplementary Table 1. Clinical features of control and ALS subjects. De-identified specimens were provided by the Department of Veterans Affairs Biorepository.

Control and ALS patients (from VA Biorepository) clinical features						
	Case #		Age	Gender	Ethnicity	PMI (cr)[b] (hrs)
Control	C-1	090015	66	M	Hispanic/Latino	<4.0
	C-4	110006	68	M	White	1.5
ALS	P-1	100031	76	M	White	3.25
	P-2	100034	62	M	African American	1.66
	P-3	100039	50	M	White	5.0
	P-4	110007	80	M	White	1.25
	P-5	110009	84	M	White	3.58
	P-8	120011	58	F	White	1.0
	P-9	120018	83	M	African American	0.25
	P-10	120021	70	M	White	0.5

Supplementary Table 2. SNP analysis of ALS patient derived FUS mutant iPSC lines.

	Exp 12	Exp23	Exp 23
	FUS P525L (fibroblasts)	FUS P525L (iPSCs)	FUS L525P (iPSCs)
SNP	allele1/allele2	allele1/allele2	allele1/allele2
C___1563023_10	VIC/FAM	VIC/FAM	NOAMP
C___1801627_20	VIC/FAM	VIC/FAM	VIC/FAM
C___2728408_10	FAM/FAM	FAM/FAM	FAM/FAM
C___1250735_20	VIC/FAM	VIC/FAM	VIC/FAM
C__15935210_10	VIC/FAM	VIC/FAM	VIC/FAM
C___7431888_10	VIC/VIC	VIC/VIC	VIC/VIC
C___3227711_10	VIC/VIC	VIC/VIC	VIC/VIC
C___1902433_10	VIC/FAM	VIC/FAM	VIC/FAM
C__30044763_10	FAM/FAM	FAM/FAM	FAM/FAM
C__31386842_10	FAM/FAM	FAM/FAM	FAM/FAM
C__33211212_10	FAM/FAM	FAM/FAM	FAM/FAM
C__26524789_10	FAM/FAM	FAM/FAM	FAM/FAM
C__11821218_10	VIC/FAM	VIC/FAM	NOAMP
C_____43852_10	VIC/FAM	VIC/FAM	VIC/FAM
C___1670459_10	FAM/FAM	FAM/FAM	FAM/FAM
C___8924366_10	VIC/VIC	VIC/VIC	VIC/VIC
C___1007630_10	VIC/VIC	VIC/VIC	VIC/VIC
C__11522992_10	FAM/FAM	FAM/FAM	NOAMP
C___7421900_10	VIC/FAM	VIC/FAM	VIC/FAM
C__10076371_10	VIC/VIC	VIC/VIC	VIC/VIC
C__26546714_10	VIC/FAM	VIC/FAM	NOAMP
C___1122315_10	VIC/VIC	VIC/VIC	VIC/VIC
C__27402849_10	VIC/FAM	VIC/FAM	VIC/FAM
C___7457509_10	VIC/FAM	VIC/FAM	VIC/FAM
C__29619553_10	VIC/FAM	VIC/FAM	NOAMP
C__11710129_10	VIC/FAM	VIC/FAM	VIC/FAM
C___2953330_10	VIC/FAM	VIC/FAM	VIC/FAM
C___1027548_20	VIC/FAM	VIC/FAM	VIC/FAM
C___8850710_10	VIC/FAM	VIC/FAM	VIC/FAM
C___1083232_10	VIC/VIC	VIC/VIC	VIC/VIC
C__16205730_10	FAM/FAM	FAM/FAM	FAM/FAM
C___8938211_20	FAM/FAM	FAM/FAM	FAM/FAM
