# Supplementary material for: Mutant FUS causes DNA ligation defects to inhibit oxidative damage repair in Amyotrophic Lateral Sclerosis
Source: Nat Commun. 2018 Sep 11;9:3683. doi: 10.1038/s41467-018-06111-6 (PMC6134028; doi:10.1038/s41467-018-06111-6)
Supplement: Supplementary file 3 — Description of Additional Supplementary Files [file 41467_2018_6111_MOESM3_ESM.pdf]

Legends for Supplementary Movie files:

Supplementary Movie 1. Recruitment of GFP-FUS WT at laser ablation track by live cell imaging. HEK293 cells were subjected to laser ablation 48h after GFP-FUS WT plasmid transfection, and the recruitment of GFP fluorescence at laser tracks was monitored by live cell imaging every 15 seconds for a period of 5 min.

Supplementary Movie 2. Recruitment of GFP-FUS R521H at laser ablation track by live cell imaging. HEK293 cells were subjected to laser ablation 48h after GFP-FUS R521H plasmid transfection, and recruitment of GFP fluorescence at laser tracks was monitored by live cell imaging every 15 seconds for a period of 5 min.

Supplementary Movie 3. Recruitment of GFP-FUS R521C at laser ablation track by live cell imaging. HEK293 cells were subjected to laser ablation 48h after GFP-FUS R521C plasmid transfection, and the recruitment of GFP fluorescence at laser tracks was monitored by live cell imaging every 15 seconds for a period of 5 min.

Supplementary Movie 4. Recruitment of GFP-XRCC1 at laser ablation track by live cell imaging. HEK293 cells were subjected to laser ablation 48h after GFP-XRCC1 plasmid transfection, and the recruitment of GFP fluorescence at laser tracks was monitored by live cell imaging every 15 seconds for a period of 5 min.

Supplementary Movie 5. Recruitment of GFP-XRCC1 at laser ablation track by live cell imaging in FUS knockdown cells. HEK293 cells were subjected to laser ablation 48h after GFP-XRCC1 and FUS shRNA co-transfection, and the recruitment of GFP fluorescence at laser tracks was monitored by live cell imaging every 15 seconds for a period of 5 min.

Legends for Supplementary Data file:

Supplementary Data 1. List of proteins isolated with FUS immunoprecipitates, identified by mass spectroscopy. SH-SY5Y cells were treated by GO (100ng/ml) for 1h, and the total lysate were extracted for co-IP by incubating with FUS antibody, followed by running 4–12% Bis-Tris gel. Gel bands were then excised and subjected to mass spectrometry analysis.
